# Continuous serratus anterior plane block for postoperative analgesia following lung transplantation via anterolateral incision: a pilot study

**DOI:** 10.3389/fmed.2024.1438580

**Published:** 2024-09-18

**Authors:** Ge Luo, Tingting Ni, Xinchen Tao, Jie Xiao, Yuanyuan Yao, Man Huang, Jingyu Chen, Min Yan

**Affiliations:** ^1^Department of Anesthesiology, The Second Affiliated Hospital of Zhejiang University School of Medicine, Hangzhou, China; ^2^Department of General Intensive Care Unit, The Second Affiliated Hospital of Zhejiang University School of Medicine, Hangzhou, China; ^3^Department of Lung Transplantation, The Second Affiliated Hospital of Zhejiang University School of Medicine, Hangzhou, China

**Keywords:** lung transplantation, serratus anterior plane block, numerical rating scale, morphine equivalents dose, analgesia

## Abstract

**Background:**

Unilateral or bilateral anterolateral thoracotomy May lead to severe acute pain in lung transplantation (LTx). Although serratus anterior plane block (SAPB) is apparently effective for pain control after open thoracic surgery, there remains a lack of evidence for the application of SAPB for postoperative analgesia after LTx.

**Objective:**

In this case series pilot study, we describe the feasibility of continuous SAPB after lung transplantation and provide a preliminary investigation of its safety and efficacy.

**Methods:**

After chest incisions closure was complete, all patients underwent ultrasound-guided SAPB with catheter insertion. Numerical rating scale (NRS), additional opioid consumption, time to endotracheal tube removal, ICU length of stay, and catheter-related adverse events were followed up and recorded for each patient within 1 week after the procedure.

**Results:**

A total of 14 patients who received LTx at this center from August 2023 to November 2023 were included. All patients received anterolateral approaches, and 10 (71.4%) of them underwent bilateral LTx. The duration of catheter placement was 2 (2–3) days, and the Resting NRS during catheter placement was equal to or less than 4. A total of 11 patients (78.6%) were supported by extracorporeal membrane oxygenation (ECMO) in LTx, whereas 8 patients (57.1%) removed the tracheal tube on the first day after LTx. Intensive care unit (ICU) stay was 5 (3–6) days, with tracheal intubation retained for 1 (1–2) days, and only one patient was reintubated. The morphine equivalent dose (MED) in the first week after LTx was 11.95 mg, and no catheter-related adverse events were detected.

**Limitations:**

We did not assess the sensory loss plane due to the retrospective design. In addition, differences in catheter placement time May lead to bias in pain assessment.

**Conclusion:**

Although continuous SAPB May be a safe and effective fascial block technique for relieving acute pain after LTx, it should be confirmed by high-quality clinical studies.

## Introduction

It is proposed that post thoracotomy pain control in lung transplantation (LTx) exerts a direct impact on short and long-term outcomes (1). Additionally, acute pain from anterolateral incisions, a common surgical incision in lung transplantation, can be severe due to the effects of skin incisions, muscle splitting, rib retraction and dislocation, pleural irritation, and intercostal nerve manipulation (2). Adequate pain management can crucially promote early extubation and early mobility and reduce intensive care unit (ICU) length of stay and mortality, in addition to reducing the incidence of chronic postoperative pain (CPSP) (3–5). Therefore, managing postoperative pain is a crucial and challenging issue in the perioperative management of LTx.

Thoracic epidural analgesia (TEA) and thoracic paravertebral block (TPVB) are traditional analgesic techniques for thoracic surgery (6, 7). However, considering the clinical challenges occasioned by systemic heparinization and the high failure rate, more safe and effective block technique is urgently required. Serratus anterior plane block (SAPB) is a typical Fascia Plane block that provides analgesia for the anterolateral chest wall. Compared with conventional blocking methods, SAPB is safer and exhibits a higher operational success rate, in addition to reducing the risk of hemorrhage and hemodynamic fluctuations, especially in patients requiring extracorporeal life support (8). Therefore, SAPB May be a potentially useful alternative. Currently, it has been widely utilized in thoracotomy (9), video-assisted thoracoscopic surgery (VATS) (10), and breast surgery (11). However, there are few studies for the application of SAPB in analgesia after LTx (12, 13); thus, the procedure lacks the support of more clinical evidence. In this case series pilot study, we aim to describe the feasibility of continuous SAPB after LTx, and to provide a preliminary investigation of its safety and efficacy.

## Methods

### Patients

A total of 14 patients who underwent continuous SAPB at our center from August 2023 to November 2023 were included. This study received ethical approval from the Institutional Research Board (IRB) in accordance with local Chinese protocols in accordance with guidelines of the Declaration of Helsinki. Written informed consent was waived due to the retrospective design. All patients had received unilateral or bilateral anterolateral thoracotomies in the supine position.

### Serratus anterior plane block procedure

**Figure 1:**
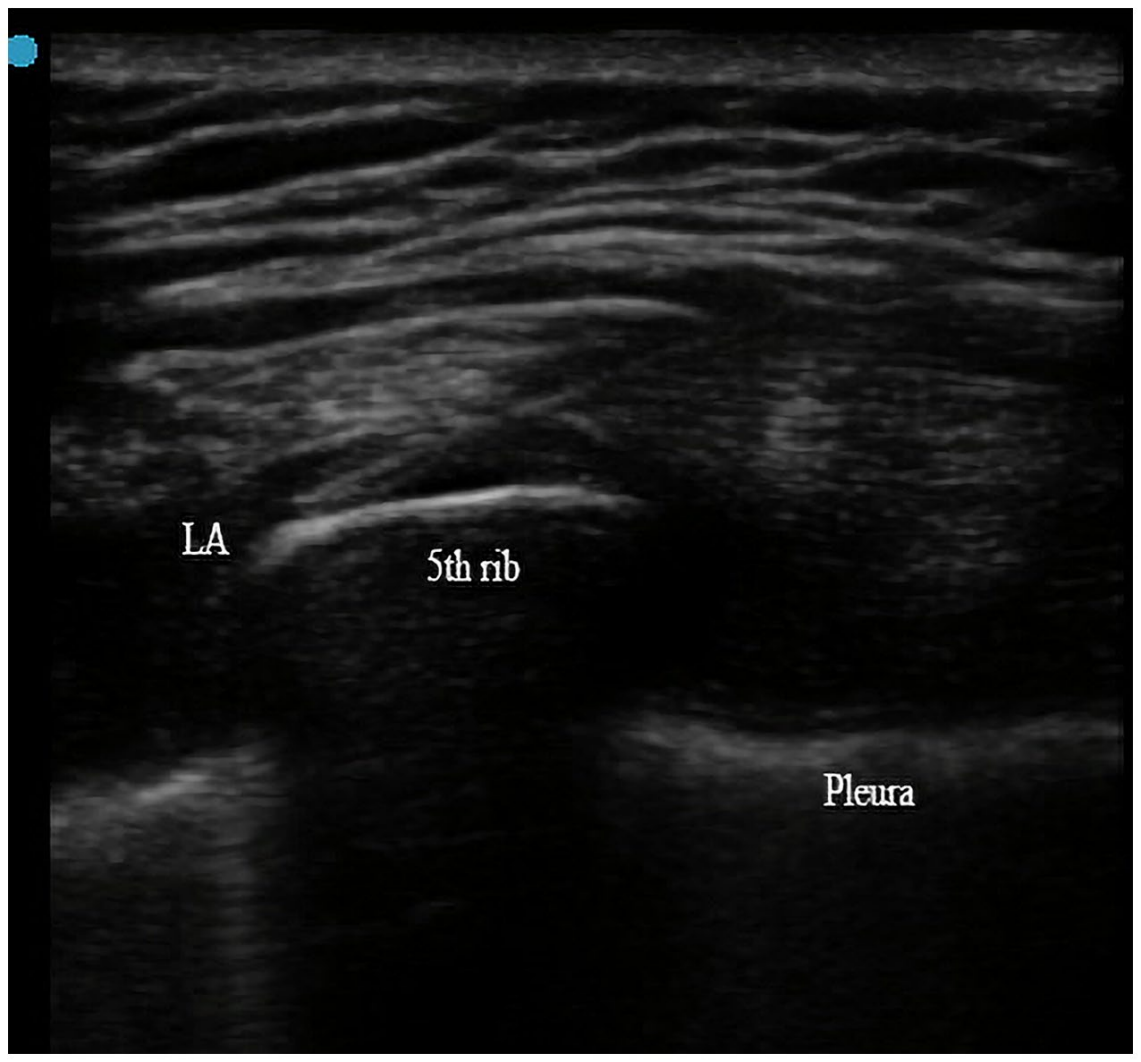
Local analgesic (LA) was injected into the plane below the serratus anterior muscle and above the fifth rib with the ultrasound guidance.

The height and inclination of the bed should be adjusted before the procedure to fully expose the lateral chest wall, and the operator should complete the plane block in a completely aseptic environment with ultrasound guidance. The continuous SAPB was performed by an attending anaesthesiologist who had experience with the ultrasound-guided fascial plane blocks after surgery in the operation room. The range of the anterolateral incision extends from the fourth or fifth intercostal space at the mid-axillary line to the same side of the parasternal region. A 38x high frequency (13–6 MHz) linear array transducer (FUJIFILM SonoSite, Inc., Bothell, WA) was placed in a sagittal plane, and the ribs were counted inferiorly and laterally, until the fifth rib was identified in the midaxillary line; subsequently, the rib, intercostales externi, and serratus muscles were identified. The tip of the 18G puncture needle (Contiplex D, B. Braun Melsungen AG, Germany) was pushed into the plane below the serratus anterior muscle and above the fifth rib; subsequently, 0.2% ropivacaine (Naropin, Astra Zeneca AB, Sweden) 20 mL was injected once (Figure 1). When local anesthetics were observed to spread optimally in the connective tissue plane, the puncture needle was subsequently removed and the catheter indwelled (Contiplex D, B. Braun Melsungen AG, Germany). The length of catheter retention should be 2–3 cm beyond the tip of the cannula. After identifying the catheter position by ultrasound and confirming the plane of drug diffusion, sterile dressings were covered. The process of continuous SAPB on the other side was the same as before. A background dose of 5 mL/h (set at 8 mL/h in single LTx) and a single injection of 10 mL of 0.2% ropivacaine at 4 h intervals were utilized for postoperative pain management. The target for catheter placement was 72 h.

### Postoperative assessment

All patients were admitted to the ICU with endotracheal tube *in situ*. Intravenous infusion of midazolam (0.08–0.10 mg/kg/h), propofol (0.80–2.50 mg/kg/h), and remifentanil (0.05–0.10 μg/kg/min) was utilized to maintain sedation and analgesia, with doses adjusted according to the Richmond Agitation-Sedation Scale (RASS) score and pain score. ECMO withdrawal and the decision to extubate was conducted after comprehensive evaluation by the physician. Pain assessment on the day of extubation begins at least 4 h after the infusion of anesthetics was stopped. Postoperative follow-up was performed by two researchers who were not involved in the data analysis process. The primary outcome was pain score within 7 days after surgery, and the secondary outcomes included additional opioid consumption, postoperative mechanical ventilation (MV) time, ICU length of stay, and catheter-related complications. We utilized 0–10 numerical rating scale (NRS) to assess the static (quiet, breath at rest) and dynamic (cough) pain scores (0 indicates no pain, whereas 10 indicates intolerable pain). Multi-modal analgesia comprised continuous SAPB, PCIA (hydromorphone) and other analgesics (dezocine, tramadol, and durogesic) which used for rescue analgesia. If NRS exceeded 3 or the patient required, PCIA or other analgesics were administered as a rescue analgesia. Additional opioid consumption was calculated from the first day after extubation, which included hydromorphone in the patient controlled intravenous analgesia (PCIA) devices and other analgesics for rescue analgesia. The calculation of morphine equivalents dose (OME) was based on the recommendation of Willenbrink. Any related complication such as exudates, catheter migration, and hematoma during catheter placement were recorded.

### Statistical analysis

The Shapiro–Wilk test was applied to assess the distribution of data and applicability of parametric tests. Data that met normal distribution criteria were described using mean ± standard deviation (SD), whereas non-normally distributed data were described using the median (IQR). Categorical data were depicted as *n* (%). *p*-value <0.05 was considered statistically significant.

## Results

A total of 14 patients who received LTx at the Second Affiliated Hospital of Zhejiang University School of Medicine from August 2023 to November 2023 were included. In this cohort, the median age of patients was 59 (52.25–60.75). Twelve (85.71%) of the 14 patients were male, and 2 (14.29%) were female. The most common indications for transplant were idiopathic pulmonary fibrosis (IPF) (42.86%), and chronic obstructive pulmonary disease (COPD) (35.71%). ECMO was utilized in 11 (78.57%) patients during LTx: the indication for ECMO was severe preoperative pulmonary hypertension or intraoperative hemodynamic instability on one-lung ventilation or during pulmonary artery clamping. All patients have received anterolateral approaches, and 10 (71.4%) of them underwent bilateral LTx. Table 1 illustrates the patients’ characteristics and surgical details.

**Table 1:** Characteristics of 14 patients who underwent LTx.

ID	Age (y)	Gender	Diagnose	Surgical type	Surgical approach	ECMO
1	60	Male	IPF	Single LTx	Supine	Yes
2	51	Female	Bronchiectasis	Double LTx	Supine	Yes
3	23	Female	BOS	Double LTx	Supine	Yes
4	61	Male	IPF	Double LTx	Supine	Yes
5	60	Male	COPD	Double LTx	Supine	Yes
6	58	Male	IPF	Single LTx	Supine	Yes
7	61	Male	Pneumoconiosis	Single LTx	Supine	Yes
8	44	Male	IPF	Double LTx	Supine	Yes
9	56	Male	IPF	Single LTx	Supine	Yes
10	59	Male	COPD	Double LTx	Supine	Yes
11	59	Male	COPD	Double LTx	Supine	No
12	49	Male	COPD	Double LTx	Supine	No
13	70	Male	IPF	Double LTx	Supine	Yes
14	63	Male	COPD	Double LTx	Supine	No

LTx, lung transplantation; ECMO, extracorporeal membrane oxygenation; IPF, idiopathic pulmonary fibrosis; BOS, bronchiolitis obliterans syndrome; COPD, chronic obstructive pulmonary disease.

**Figure 2:**
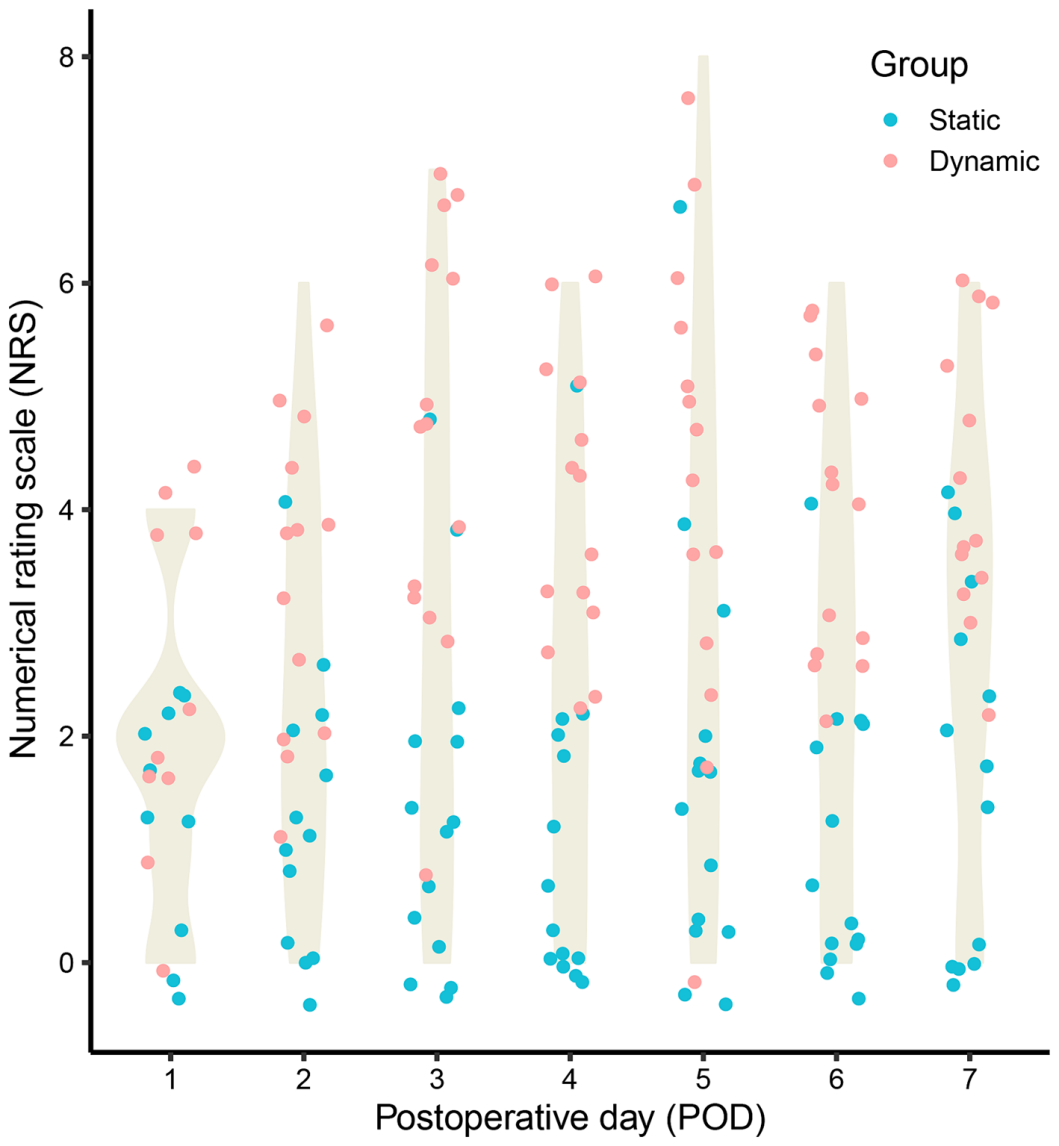
The static and dynamic numerical rating scale (NRS) of 14 patients within 7 days after lung transplantation (LTx).

Four patients remained in moderate and deep sedation on post operative day 1 (POD1), and one patient was intubated on POD7 after operation. Generally, a NRS score of less than 4 indicates satisfactory pain control. The duration of catheter placement was 2 (2–3) days, during which maximum daily pain at rest did not exceed 4 measured using a 0–10 NRS. Furthermore, the maximum daily pain at rest was a median of 0.5–2 for the entire follow-up period. Active NRS was below or equal to 4 within the first 2 days of catheter placement in all patients except three (Number 5, 8 and 9) of them. Maximum pain each day with movement was a median of 2 and 4 on POD1 and POD2, respectively, but subsequently rose above 4 for the remaining time points. Table 2 indicates the NRS of 14 patients within 7 days after LTx. The visualization results were presented in Figure 2.

**Table 2:** The NRS of 14 patients within 7 days after LTx.

ID	POD1		POD2		POD3		POD4		POD5		POD6		POD7	
	Static	Dynamic	Static	Dynamic	Static	Dynamic	Static	Dynamic	Static	Dynamic	Static	Dynamic	Static	Dynamic
1	Sedation		1	4	1	6	0	4	0	4	0	3	0	3
2	0	2	3	3	1	3	0	3	1	3	0	3	0	3
3	Sedation		Sedation		2	7	2	6	3	7	2	5	2	5
4	2	4	1	3	0	3	1	3	2	6	1	3	4	5
5	2	4	2	5	2	5	2	4	2	4	1	4	4	6
6	1	2	1	2	1	3	0	2	0	2	0	4	1	4
7	2	2	1	2	1	3	1	3	1	2	0	3	3	3
8	2	4	2	6	2	5	2	6	2	5	4	5	3	4
9	Sedation		4	5	5	6	5	5	4	6	2	6	2	6
10	0	1	0	1	0	1	0	2	0	0	0	2	0	2
11	2	4	0	4	0	7	0	3	0	5	0	5	0	4
12	1	2	0	2	0	4	0	4	7	8	2	3	Reintubation	
13	Sedation		2	4	0	5	0	5	0	4	0	4	0	4
14	0	0	0	4	4	7	2	5	2	5	2	6	2	6

NRS, numerical rating scale; LTx, lung transplantation; POD, postoperative day.

Table 3 listed the results of outcomes assessment. The length of ICU stay was 5 (3–6) days, with tracheal intubation retained for 1 (1–2) days, and only one patient was reintubated. A high percentage of patients met the respiratory goals soon after surgery (extubated): 57.1% within 24 h after surgery (8/14) and 92.8% within 48 h after surgery (13/14). The duration of chest tube placement was 5 (3–5.75) days. The additional opioids that were utilized was 11.95 (2.25–84.88) mg (morphine equivalents dose [MED]) in the first week after tracheal tube removal, and no SAPB catheter-related adverse events (e.g., infection, exudation, and dislodgement) were detected.

**Table 3:** Outcomes assessment for 14 patients after LTx.

Outcomes	All patients (*n* = 14)
Length of ICU stay, median(IQR), d	5 (3–6)
Duration of mechanical ventilation median (IQR), d	1 (1–2)
Reintubation, *n* (%)	1 (7.14)
Extubation within 24 h, *n* (%)	8 (57.1)
Extubation within 48 h, *n* (%)	13 (92.8)
Duration of chest tube placement, median (IQR), d	5 (3–5.75)
Morphine equivalents dose, median (IQR), mg	11.95 (2.25–84.88)
Catheter-related adverse events, *n* (%)	0 (0)

LTx, lung transplantation; ICU, intensive care unit; IQR, interquartile range.

## Discussion

Our findings suggest that continuous SAPB is a potentially safe and effective technique for managing acute pain after LTx via the anterolateral approach. Resting NRS scores remained at or below 4 during catheter placement, and opioid consumption was relatively low (11.95 mg in the first week), indicating good pain control with minimal opioid use. Additionally, no catheter-related complications were observed.

Acute pain after LTx is a crucial concern. Several factors including surgical incision, disruption of ribs and intercostal nerves, and the placement of chest drainage tubes are potential sources of postoperative pain (14). Uncontrolled pain May hinder a powerful cough, occasion effective pulmonary function exercise, induce graft expansion and prolonged hospital stay, and increase the consumption of opioids and the risk of postoperative pulmonary complications (PPCs) (4, 12). Optimizing acute pain management after LTx can enhance pulmonary mechanics and reduce the risk of CPSP (15). There are four surgical approaches in LTx including anterolateral thoracotomy, posterolateral thoracotomy, clamshell incision, or sternotomy. Considering the surgical approach, extracorporeal life support (ECLS), and the etiology of LTx, it was difficult to formulate a standardized analgesic strategy.

TEA is considered the gold standard for postoperative analgesia in thoracic surgery, and TPVB has already been revealed to be no less effective than TEA for analgesia (16). However, the high difficulty and failure rate May limit their application. In addition, considering the requirement of systemic anticoagulants for ECLS, epidural hematoma (SEH) and unpredictable bleeding due to deep puncture May lead to severe consequences.

SAPB is a novel fascial plane block technique that targets the lateral cutaneous branch of the T2–T9 intercostal nerve (13). Currently, the efficiency of SAPB in postoperative analgesia after thoracotomy has been confirmed in many studies (17, 18). However, evidence for the application of SAPB in LTx is rare. In addition, there remains a lack of evidence based on Asian populations: ethnic differences are not considered. Therefore, this study reported a series of data on the application of continuous SAPB as an analgesic technique in 14 patients with end-stage lung disease who underwent LTx.

Herein, the median duration of catheter placement was 2 days, and all patients exhibited static NRS of less than 4, whereas only 3 patients exhibited dynamic NRS more than 4 during catheter placement. Total MED within 1 week of catheter removal was 11.95 (2.25–84.88) mg. In a previous retrospective study, Lenz et al. (19) explored the effects of TEA, TPVB, and systemic analgesia (SA) in postoperative analgesia after LTx. Patients receiving TPVB exhibited a median MED of more than 200 mg on POD1, and some patients continued to receive surprisingly high levels of additional opioid use up to POD7. Despite the differences in analgesic strategy, it is undeniable that the utilization of opioids in the research of Lenz et al. far exceeds the opioid-utilization levels utilize herein. High remedial analgesic usage after SAPB has been found in other studies (13, 20), a discrepancy that is attributable to numerous reasons. First, the analgesic strategy included strong opioids such as Remifentanil, Fentanyl, and Durogesic. However, in this study, only 1 patient utilized Durogesic, and the majority of patients received analgesics such as hydromorphone, dizocin, and tramadol. Second, owing to the limitations of retrospective studies, the utilization of analgesics May not have been strictly regulated and controlled. In addition, differences between ethnicities May be a crucial factor that cannot be ignored. According to the existing literatures, the prevalence of moderate to severe postoperative pain is 86 and 70% in the United States and Europe, respectively (21, 22). A study based on the Chinese population reported only 48.7% (23), which is significantly lower than those reported in America and Europe. Some studies also noted that that Asian populations exhibit more optimal tolerance for pain and require less analgesics than European populations. Goh et al. also found that Chinese surgical patients exhibited a higher tolerance for pain compared to other Asian populations (24).

However, we should still emphasize that the high tolerance for pain does not imply the non-essentiality of acute pain control; contrastingly, uncontrolled postoperative pain can directly limit the patient’s initiative in respiratory and functional exercises. We found that in 10 patients, the tracheal tube was removed on POD1, and that the median duration of drainage tube placement was 5 days, with the length of ICU stay of 5 (3–6) days. This result also indicated that the implementation of continuous SAPB for early control of acute pain May provide patients with early extubation and effective breathing exercises. Even though a previous study has demonstrated that continuous SAPB can improve lung function and reduce postoperative PPCs in postoperative lung cancer patients (25). We did not dynamically monitor the patients’ postoperative lung function variables (LFVs), including FEV1, FVC, and FEV1/FVC.

As a superficial fascial plane block, the safety of SAPB was also preliminarily confirmed. In 14 patients, despite ECMO usage attaining 78.6%, no catheter-related adverse events such as exudates, migration or detachment, and hematoma around the site of puncture were detected during catheter placement. Anderson et al. (12) also noted that because SAPB was not a method for direct nerve block, the risk of neural injection was low. Compared with TEA, we do not need to focus on the increased risk of potential bleeding occasioned by systemic heparinization due to ECLS. In addition, SAPB can more easily identify the target under ultrasound guidance, with fewer blood vessels and lower risk of hematoma; therefore, it is apparent that SAPB exhibits more optimal safety and operability.

However, in the performance of the analgesic strategy, we still found some potential problems. First, contradictory results have been obtained in clinical studies involving the application of superficial and deep SAPB (20, 26), and there is no apparent evidence to prove that that deep SAPB exhibits more optimal results in pain management after LTx. In fact, under the guidance of ultrasound, anesthesiologists can locate the ribs and serratus anterior muscle and identify the deep serratus anterior muscle plane more quickly and accurately, which would shorten the time of puncture to a great extent, and bears potential value for realizing the rapid transport of patients. In addition, some studies have indicated that local anesthetics can easily obtain satisfactory diffusion in the deep serratus anterior plane, which May lead to a more optimal analgesic effect (20, 27). On the other hand, deep SAPB May be more stable for catheter anchoring (13). Second, the timing of catheter placement remains inconclusive. The placement of the catheter before operation (28) is limited by factors such as the possibility of surgery cancellation, the time limit at the beginning of the surgery, the impact on the surgical process, the cutting of the catheter, and the selection of multiple incisions. Although we have also considered continuous SAPB prior to tracheal catheter removal, it is difficult to meet strict sterility requirements using the aforementioned method, which May pose additional risks under palliative sedation. The target time for catheter placement was set at 72 h. Although prolonged catheter placement May provide additional benefits for acute pain management, it May also increase the possibility of infection and catheter migration (8). In addition, all patients included herein underwent unilateral or bilateral antero-lateral thoracotomy, The application of SAPB targeting the cutaneous lateral branch of the intercostal nerve in anterolateral thoracotomy is reasonable. However, in the postoperative pain management of patients undergoing sternotomy, the utilization of continuous SAPB as component of multimodal analgesia is apparently unpersuasive. Several research have already found that parasternal intercostal nerve block, an approach of blocking the cutaneous anterior branch of the intercostal nerve, can alleviate postoperative pain and reduce the consumption of opioids in cardiac surgery with sternotomy (29, 30). However, there was no direct evidence for the benefits of parasternal intercostal nerve block in LTx.

This study exhibited the following limitations. First, we did not evaluate the plane of loss of sensation; thus, we were uncertain about the range of deep SAPB. Second, the participation of different follow-up researchers May lead to bias in the outcome assessment. Third, the use of rescue analgesics was inconsistent, which May lead to variability in the analgesic outcomes. However, this approach reflects the personalized pain management commonly employed in clinical practice, where pain management are tailored to each patient’s condition to optimize therapeutic outcomes. Fourth, although the target for catheter placement was set at 3 days, the majority of patients with catheter for only 2 days and differences in the duration of catheterization in this cohort May influence the subsequent trends of pain. Finally, the lack of a control group, single-center setting, and small sample size limit the generalizability of our findings. Further multicenter studies with larger sample sizes and randomized controlled trials are required to confirm these results.

## Conclusion

Continuous SAPB May be a safe and effective fascial plane block technique for relieving acute pain after LTx via anterolateral approach. However, owing to the characteristic and complexity of LTx, more high-quality research focused on the technical details is required.

## Data Availability

The raw data supporting the conclusions of this article will be made available by the authors, without undue reservation.
