# Supplementary figures and images for: Continuous serratus anterior plane block for postoperative analgesia following lung transplantation via anterolateral incision: a pilot study
Source: Front Med (Lausanne). 2024 Sep 18;11:1438580. doi: 10.3389/fmed.2024.1438580 (PMC11445064; doi:10.3389/fmed.2024.1438580)

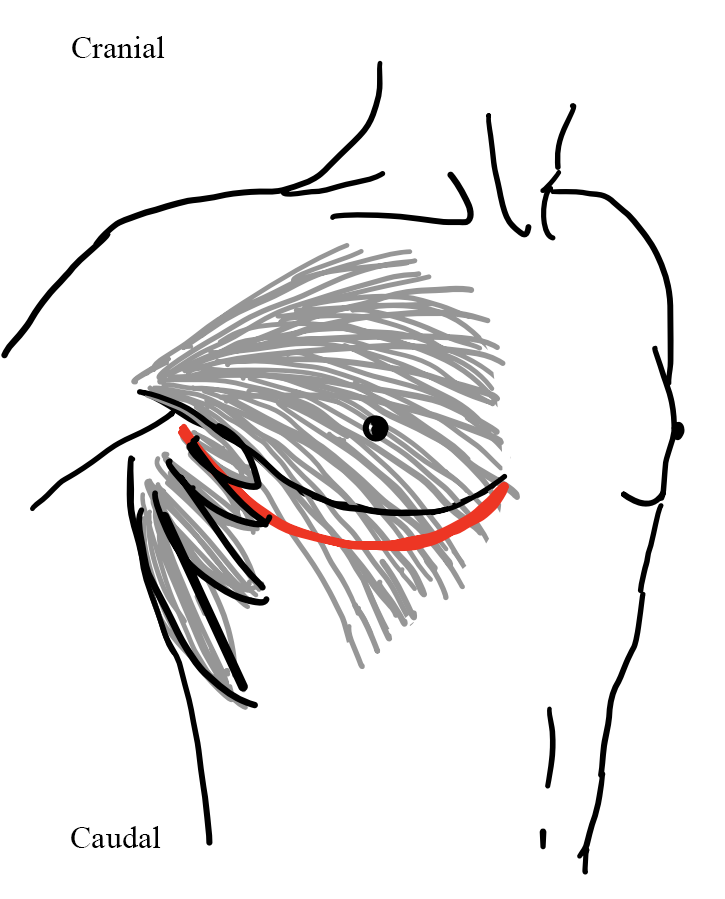

Supplement: Supplementary file 1 [file Image_1.TIF]
